# Prognostic factors in epithelial ovarian cancer: A population-based study

**DOI:** 10.1371/journal.pone.0194993

**Published:** 2018-03-26

**Authors:** Lin-Chau Chang, Chih-Fen Huang, Mei-Shu Lai, Li-Jiuan Shen, Fe-Lin Lin Wu, Wen-Fang Cheng

**Affiliations:** 1 School of Pharmacy, College of Medicine, National Taiwan University, Taipei, Taiwan; 2 Department of Pharmacy, National Taiwan University Hospital, Taipei, Taiwan; 3 Institute of Epidemiology and Preventive Medicine, College of Public Health, National Taiwan University, Taipei, Taiwan; 4 Graduate Institute of Clinical Pharmacy, College of Medicine, National Taiwan University, Taipei, Taiwan; 5 Department of Obstetrics and Gynecology, College of Medicine, National Taiwan University, Taipei, Taiwan; 6 Graduate Institute of Oncology, College of Medicine, National Taiwan University, Taipei, Taiwan; 7 Graduate Institute of Clinical Medicine, College of Medicine, National Taiwan University, Taipei, Taiwan; University of South Alabama Mitchell Cancer Institute, UNITED STATES

## Abstract

The overall survival (OS) of patients with ovarian cancer is poor while epithelial ovarian cancer (EOC) is the most lethal gynecologic cancer. The aim of the present study was to evaluate the clinico-pathologic characteristics, especially the prognostic factors, for patients with epithelial ovarian cancer (EOC) in Taiwan. Information about newly diagnosed patients with EOC from 2009 to 2012 was retrieved from the database of the Taiwan Cancer Registry. Data from 2009 to 2013 for the respective cases from the claims database of Taiwan’s National Health Insurance and National Death Registry were then retrieved. Potential prognostic factors were analyzed. The mean age at diagnosis of the 2,498 patients was 52.8 years. Serous carcinoma and clear cell carcinoma were diagnosed in 43.3% and 22.8% of the total patients, respectively. For patients with early-stage disease, taxane-based adjuvant chemotherapy, stage I, and younger age at diagnosis led to better overall survival (p = 0.030, p = 0.002, p<0.001, respectively) in multivariable analysis. For advanced-stage patients, histology (endometrioid type), taxane-based adjuvant chemotherapy, stage, and age at diagnosis had a significant impact on OS (p<0.001, p = 0.020, p<0.001, p<0.001, respectively). In conclusion, taxane-based chemotherapy impacts the outcome of patients with EOC. Personalized medicine may be needed for different histological types of EOC because of their different outcomes.

## Introduction

Ovarian cancer is the seventh most common cancer in women worldwide [1]. Due to difficulties in early detection, diagnosis, and treatment, the overall survival (OS) of patients with ovarian cancer is poor [2, 3]. Epithelial ovarian cancer (EOC) is the most lethal gynecologic cancer [1, 2]. Some factors that could impact OS may include cancer stage, histological type, early recognition, patient management, age, comorbidities, and type of hospital [3–8]. Current recommended frontline therapy remains the same for epithelial subtypes, which consists of upfront surgery followed by chemotherapy based on a combination of a platinum drug and paclitaxel [9]. For invasive or recurrent ovarian cancer, individualized assessments and optimized management are especially required [10, 11].

Among different histological subtypes of EOC with varying molecular, clinical, and pathological characteristics [12–16], clear cell carcinoma is suggested to be a distinct subtype and is associated with a poorer prognosis compared to other histological subtypes, such as the more common serous carcinoma, if diagnosed at an advanced stage [14–16]. However, results vary, especially for patients diagnosed at an early stage of disease [14–16]. Moreover, the more common high-grade and the less common low-grade serous carcinomas are two different entities with divergent underlying pathogenesis, molecular events, behaviors, and disease prognoses [14, 17]. Therefore, histology-specific research is essential for facilitating individualized treatment of EOC.

Previously, Chiang et al. reported trends in the incidence and survival outcomes of women with EOC from 1979 to 2008 in Taiwan [3]. However, Chiang et al. did not have enough data to further analyze the influences of stage and chemotherapeutic regimens in patients with EOC because complete information regarding the aforementioned factors was not available in the database prior to 2008 [3]. Therefore, the aim of the present study was to investigate the clinico-pathologic characteristics of patients with EOC in terms of demographics, survival outcomes, and prognostic factors including histological types using the database from the Taiwan Cancer Registry (TCR). The study aimed to shed light on the epidemiology of EOC in Taiwan as well as provide more evidence for the importance of histology-specific considerations and treatment selection.

## Materials and methods

### Patients and data sources

**Fig 1:**
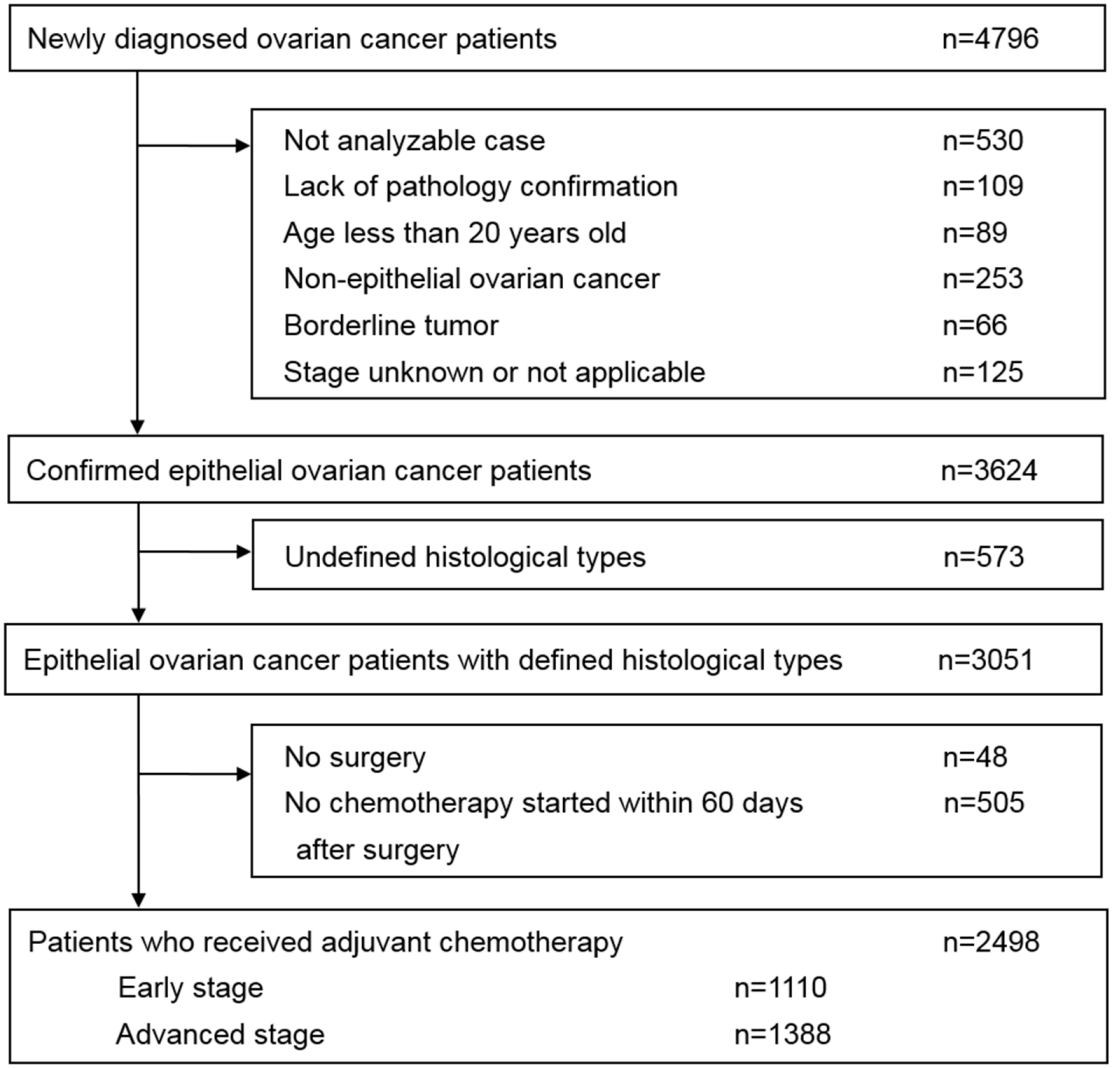
Diagram showing the inclusion and exclusion criteria for eligible patients.

Newly diagnosed patients with EOC from January 1, 2009 to December 31, 2012 were identified from the TCR database which is a population-based cancer registry established in 1979 [18]. The TCR system consists of information about patients newly diagnosed with malignant neoplasms as reported by hospitals with more than a 50-bed capacity in Taiwan [18]. It is maintained by the National Public Health Association [18]. The data from the claims database of Taiwan’s National Health Insurance (NHI) and National Death Registry from January 1, 2009 to December 31, 2013 for the respective cases were retrieved. The NHI program is a system of healthcare services founded in 1995 and is compulsory for all citizens in Taiwan [19, 20]. Therefore, the claims database contains comprehensive and important healthcare records that facilitate population-based studies [20]. The diagram showing the criteria for inclusion and exclusion of patients in the study is illustrated in Fig 1. All the data used in the present study were anonymous without any identifiable personal information and were available through formal application to the Health and Welfare Data Science Center at Ministry of Health and Welfare, Taiwan. The study protocol was qualified for exempt review by the Institutional Review Board of the National Taiwan University Hospital.

### Coding system and the histological classification of epithelial ovarian cancer

For the TCR database, the disease coding system was based on the International Classification of Diseases for Oncology, 3rd Edition (ICD-O-3) [21]. For the retrieval of cases with ovarian cancer, newly diagnosed cases with a primary cancer of the ovary, fallopian tube, and broad ligament (ICD-O-3 C56, C57.0-C57.4) were identified [22]. Among these cases, patients meeting the inclusion criteria shown in Fig 1 were eligible for the present study. The histological types included in the present study consisted of serous carcinoma (8441/3, 8460/3, 8461/3, 8442/3, 8463/3), clear cell carcinoma (8310/3, 8313/3), endometrioid carcinoma (8380/3, 8382/3, 8383/3, 8381/3, 8805/3, 8930/3), mucinous carcinoma (8470/3, 8471/3, 8480/3, 8482/3, 8472/3, 9015/3), and others including undifferentiated carcinoma (8020/3, 8021/3), malignant Brenner tumor (9000/3), and mixed cell adenocarcinoma (8323/3). The selection of morphology codes (histology/behavior grade) [21] applied herein was based on those used by Chiang et al. [3] and in the Cancer Registry Annual Report, 2012, Taiwan [22]. Patients with non-epithelial ovarian cancer inclusive of ovarian germ cell tumors and ovarian sex-cord stromal tumors, undefined histological types of ovarian cancer, or other primary tumors were excluded. In addition, all clear cell carcinomas were regarded as being of high grade. The staging system in the present article referred to the 7th Edition American Joint Committee on Cancer (AJCC) Cancer Staging [22, 23].

### The use of chemotherapy and the analysis of survival data

Chemotherapy initiated within two months after surgery was defined as adjuvant chemotherapy. While the use of taxane-based adjuvant chemotherapy such as paclitaxel was reimbursed for advanced-stage EOC, it was not reimbursed for early-stage EOC under the current NHI system. In order to investigate the impact of the use of taxanes and to minimize the interference of results due to self-subsidized treatments beyond the NHI system, searches were done for the combinatorial use of dexamethasone, anti-histamine, and H_2_-receptor antagonists with platinum-based therapy to correct for the potential underlying treatment with paclitaxel for early-stage EOC. OS was defined as the length of time from the date of surgery until the date of death or the end of observation (December 31, 2013) for patients who were still alive. Data for patients who were still alive at the end of the observation (December 31, 2013) were considered censored.

### Statistical analysis

The means or frequencies of patient characteristics and treatment modalities were compared across various subgroups. One-way analysis of variance or t-test was used for continuous variables, and chi-square test was used for categorical variables. Survival curves were estimated using the Kaplan-Meier method, and the log-rank test was used to compare differences between groups. The Cox-proportional hazards model was applied for multivariable analysis. Hazard ratios were calculated after adjustment for various factors. A logistic regression model was used to estimate the odds ratio of binary dependent variables. Two-sided p values less than 0.05 were considered statistically significant. All analyses were performed using SAS statistical software, version 9.4 (SAS Institute Inc., Cary, NC).

## Results

### Characteristics of patients with epithelial ovarian cancer

Initially, 4,796 patients with newly diagnosed ovarian cancer were identified from the TCR database. Among these patients, the demographics and characteristics of 2,498 patients who met the inclusion criteria for the present study are collectively displayed in Table 1. The mean of the age at diagnosis was 52.8 years old. Advanced stage (stages III and IV) disease was diagnosed in 55.6% of patients. The majority of patients were treated in medical centers (74.3%). Of the total patients, 43.3% were diagnosed with serous carcinoma, 22.8% with clear cell carcinoma, 17.3% with endometrioid carcinoma, 11.2% with mucinous carcinoma, and 5.4% with undifferentiated carcinoma, malignant Brenner tumor, or mixed types of adenocarcinoma. Among serous carcinoma, 83.8% were diagnosed at an advanced-stage. On the contrary, among clear cell and endometrioid carcinomas, 69.4% and 63.0% were diagnosed with early stage I and II, respectively.

**Table 1:** The demographics and clinico-pathologic characteristics of 2498 women with epithelial ovarian cancer.

		Total		Serous carcinoma		Clear cell carcinoma		Endometrioid carcinoma		Mucinous carcinoma		Others^a^	
Characteristics		n	(%)	n	(%)	n	(%)	n	(%)	n	(%)	n	(%)
**Number of patients**		2498	(100.0)	1081	(100.0)	569	(100.0)	433	(100.0)	279	(100.0)	136	(100.0)
**Age at diagnosis**^b^	Mean (SD)	52.8 (11.2)		55.8 (11.3)		50.5 (9.2)		50.7 (10.9)		49.7 (13.1)		51.6 (10.0)	
	20–39	265	(10.6)	64	(5.9)	64	(11.2)	66	(15.2)	57	(20.0)	14	(10.3)
	40–59	1622	(64.9)	652	(60.3)	419	(73.6)	289	(66.7)	163	(58.4)	99	(72.8)
	≥60	611	(24.5)	365	(33.8)	86	(15.1)	78	(18.0)	59	(21.1)	23	(16.9)
**Stage**	I	852	(34.1)	102	(9.4)	329	(57.8)	198	(45.7)	176	(63.1)	47	(34.6)
	IA/B	334	(13.4)	38	(3.5)	144	(25.3)	69	(15.9)	65	(23.3)	18	(13.2)
	IC	518	(20.7)	64	(5.9)	185	(32.5)	129	(29.8)	111	(39.8)	29	(21.3)
	II	258	(10.3)	73	(6.8)	66	(11.6)	75	(17.3)	21	(7.5)	23	(16.9)
	III	1092	(43.7)	715	(66.1)	134	(23.6)	125	(28.9)	63	(22.6)	55	(40.4)
	IV	296	(11.8)	191	(17.7)	40	(7.0)	35	(8.1)	19	(6.8)	11	(8.1)
**Grade**	Low (I/II)	703	(28.1)	203	(18.8)	0	(0.0)	282	(65.1)	170	(60.9)	48	(35.3)
	High (III)	1496	(59.9)	710	(65.7)	569	(100.0)	117	(27.0)	34	(12.2)	66	(48.5)
	Unknown	299	(12.0)	168	(15.5)	0	(0.0)	34	(7.9)	75	(26.9)	22	(16.2)
**Year of diagnosis**	2009	568	(22.7)	251	(23.2)	126	(22.1)	93	(21.5)	71	(25.4)	27	(19.9)
	2010	647	(25.9)	291	(26.9)	137	(24.1)	115	(26.6)	64	(22.9)	40	(29.4)
	2011	638	(25.5)	267	(24.7)	139	(24.4)	121	(27.9)	75	(26.9)	36	(26.5)
	2012	645	(25.8)	272	(25.2)	167	(29.3)	104	(24.0)	69	(24.7)	33	(24.3)
**Facility type**	Medical center	1857	(74.3)	827	(76.5)	440	(77.3)	308	(71.1)	177	(63.4)	105	(77.2)
	Others	641	(25.7)	254	(23.5)	129	(22.7)	125	(28.9)	102	(36.6)	31	(22.8)

SD, standard deviation; n, patient number.

^a^Including undifferentiated carcinoma, malignant Brenner tumor, and mixed cell adenocarcinoma.

^b^Age in years.

### Overall survival of patients with early-stage EOC

**Fig 2:**
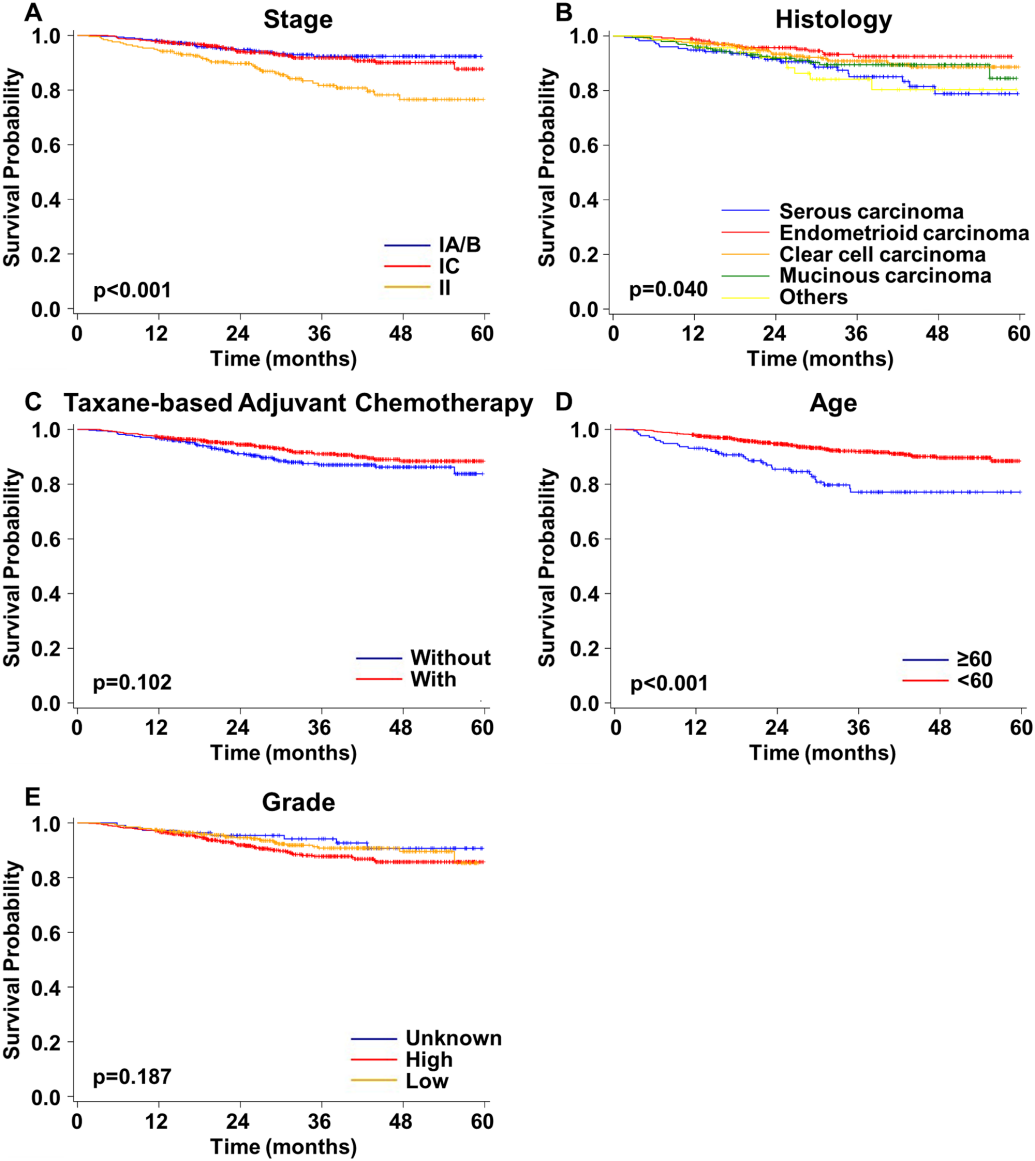
Overall survival curves for patients with early-stage epithelial ovarian cancer. **A)** Stages IA/B, IC, and II. **B)** Different histological types. **C)** Taxane- and non-taxane-based adjuvant chemotherapy. **D)** Different ages at diagnosis. **E)** Different grades.

There were 1,110 patients with early-stage EOC. Stage (p<0.001) and histological type (p = 0.040) significantly influenced OS (Fig 2A and 2B). The OS of patients with stage II was poorer than that of patients with stages IA/B or IC (Fig 2A). The OS of patients with serous carcinoma was poorer than that of patients with endometrioid, clear cell, or mucinous carcinoma (Fig 2B). The use of taxane-based adjuvant chemotherapy did not significantly alter OS (p = 0.102) (Fig 2C). The OS of patients ≥60 years was significantly poorer than that of patients <60 years (p<0.001) (Fig 2D). The histological grades did not significantly affect the OS of patients with early-stage EOC (p = 0.187) (Fig 2E).

### Overall survival of patients with advanced-stage EOC

**Fig 3:**
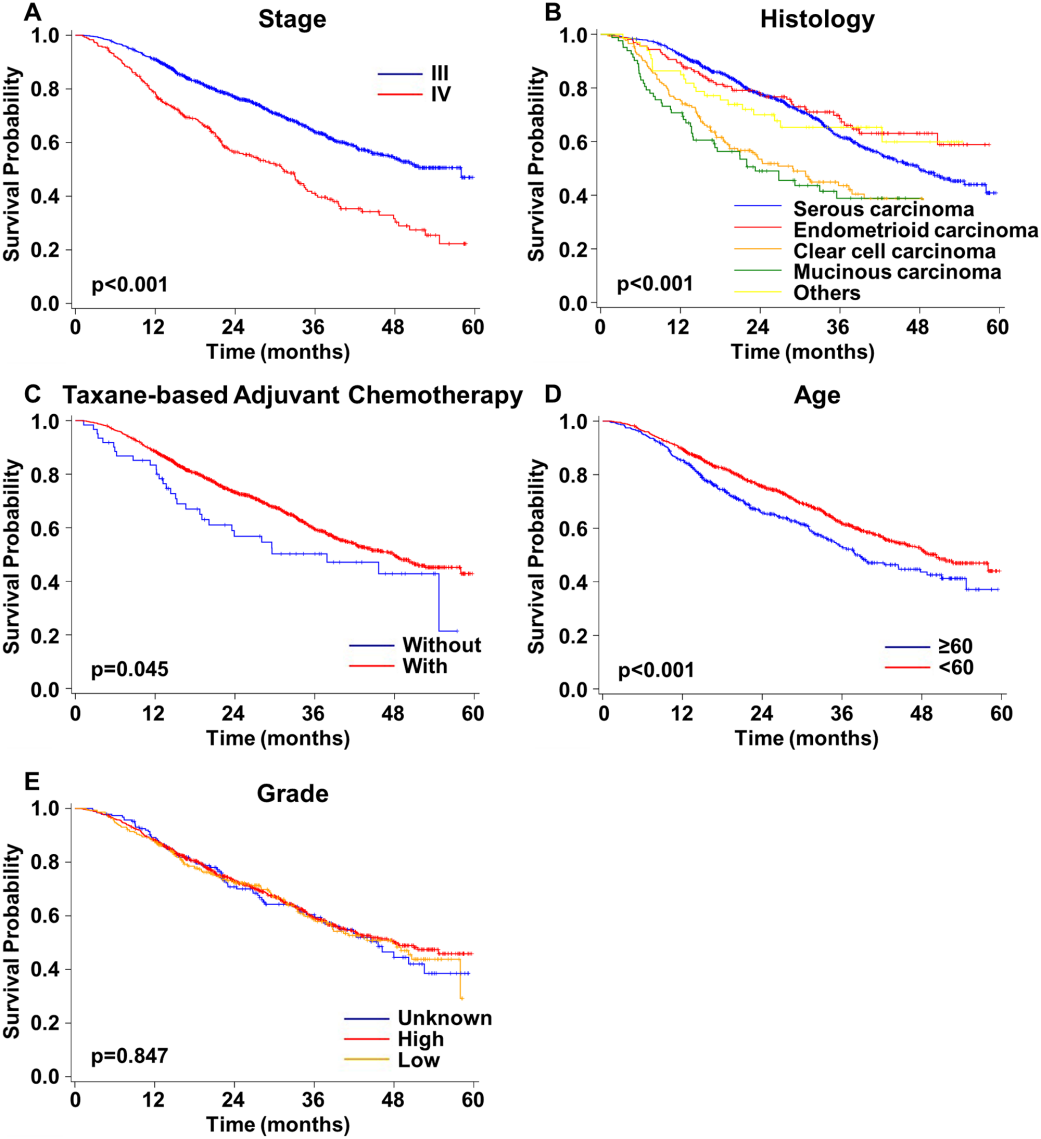
Overall survival curves for patients with advanced-stage epithelial ovarian cancer. **A)** Stages III and IV. **B)** Different histological types. **C)** Taxane- and non-taxane-based adjuvant chemotherapy. **D)** Different ages at diagnosis. **E)** Different grades.

A total of 1,388 patients with advanced-stage EOC were analyzed. The OS of patients with stage III was significantly better than that of patients with stage IV (p<0.001) (Fig 3A). The OS of patients with different histological types were also significantly different (p<0.001) (Fig 3B). Patients with clear cell or mucinous carcinoma had shorter OS than those with endometrioid or serous carcinoma (Fig 3B). The group using taxane-based adjuvant chemotherapy also had significantly better OS than the group using non-taxane-based chemotherapy (p = 0.045) (Fig 3C). The OS of patients aged ≥60 years was significantly poorer than that for patients aged <60 years (p<0.001) (Fig 3D). The histological grade did not significantly influence the OS of these patients with advanced-stage EOC (p = 0.847) (Fig 3E).

### Multivariable analyses

As shown in Table 2, taxane-based adjuvant chemotherapy (p = 0.030), stage I (p = 0.002), and a younger age at diagnosis (p<0.001; <60 years old) were three independent prognostic factors for longer survival in patients with early-stage EOC. In patients with advanced-stage EOC, having endometrioid carcinoma (p<0.001), taxane-based adjuvant chemotherapy (p = 0.020), stage III (p<0.001), and a younger age at diagnosis (p<0.001; <60 years) were prognostic factors for longer survival (Table 2).

**Table 2:** Multivariable analysis of risk factors for overall survival in patients with epithelial ovarian cancer who have early or advanced stage disease.

Variable^a^		Overall survival					
		Early stage			Advanced stage		
		AHR	95%CI	*p*	AHR	95%CI	*p*
**Histology**	Serous carcinoma	Ref.		0.208	Ref.		**<0.001**
	Clear cell carcinoma	0.73	(0.41, 1.32)		2.41	(1.88, 3.10)	
	Endometrioid carcinoma	0.64	(0.32, 1.29)		0.84	(0.61, 1.15)	
	Mucinous carcinoma	1.27	(0.62, 2.62)		2.19	(1.58, 3.03)	
	Others^b^	1.28	(0.59, 2.74)		1.13	(0.73, 1.74)	
**Adjuvant chemotherapy**	Taxane-based	Ref.		**0.030**	Ref.		**0.020**
	Non-taxane-based	1.57	(1.04, 2.37)		1.56	(1.07, 2.26)	
**Stage**	IA/IB	Ref.		**0.002**			
	IC	1.23	(0.72, 2.09)				
	II	2.45	(1.40, 4.27)				
	III				Ref.		**<0.001**
	IV				2.01	(1.66, 2.42)	
**Age at diagnosis (years)**	<60	Ref.		**<0.001**	Ref.		**<0.001**
	≥60	2.22	(1.44, 3.43)		1.37	(1.14, 1.64)	
**Grade**	Low (I/II)	Ref.		0.198	Ref.		0.268
	High (III)	1.57	(0.87, 2.83)		0.83	(0.66, 1.05)	
	Unknown	0.80	(0.36, 1.76)		0.93	(0.69, 1.25)	

AHR, adjusted hazard ratio; CI, confidence interval; Ref., reference.

^**a**^The variable, residual tumor, was included in the analysis. However, due to a significant lack of information in the database, conclusions for residual tumor may not be drawn in the present study.

^b^Including undifferentiated carcinoma, malignant Brenner tumor, mixed cell adenocarcinoma.

## Discussion

Due to the compulsory nature of the NHI program as well as the increasing completeness and quality of the TCR database, the information retrieved from the database was considered representative and useful for investigating survival outcomes in patients with EOC in Taiwan [3, 19, 20]. Although the morphology codes compiled herein may not be exactly the same as those used by Chiang et al. for the study of women with EOC from 1979 to 2008 in Taiwan [3], the results obtained in the present study were mostly in line with the trends previously observed, while more factors were discussed due to the advancement of the TCR database, especially the important findings related to the use of taxanes. Chiang et al. reported that the peak age at diagnosis changed from approximately age 60 years to 50 years from 1979 to 2008 [3], and the mean of the age at diagnosis in the present study was 52.8 years old. Meanwhile, women diagnosed with clear cell carcinoma were younger than those diagnosed with serous carcinoma (Table 1), which was consistent with many previous findings [3,14–16].

The percentage of early-stage patients (44.4%) during the study period in the present study was relatively high [5, 7, 8, 11, 15], which may be attributed to relatively early diagnosis due to the high accessibility of health examinations and easy access to ultrasonography in Taiwan [19]. Regarding the pattern of histological types, which varied across studies [5, 7, 11, 15], the percentage (43.3%) of patients with serous carcinoma in the present study was found to be similar to that of patients with serous carcinoma (41.4%) from 1979 to 2008 in Taiwan [3]. Likewise, the percentages (17.3% in the present study; 17.5% in Chiang et al. [3]) of patients with endometrioid carcinoma were also similar. The percentage (11.2%) of patients with mucinous carcinoma in the present study was less than that (24.5%) of patients with mucinous carcinoma from 1979 to 2008 in Taiwan [3], which correspond to the decreasing trend demonstrated by Chiang et al. [3]. The increasing percentage (22.8% in the present study; 13.7% in Chiang et al. [3]) of patients with clear cell carcinoma also conformed to the increasing trend found by Chiang et al. [3]. It has been reported that clear cell carcinoma was more often diagnosed in patients with early-stage disease [15, 16, 24]. In the present study, 69.4% of patients with clear cell carcinoma had early-stage disease, while only 16.2% of patients with serous carcinoma had early-stage disease (Table 1).

As for the potential prognostic factors, histological type was found to significantly influence the survival of patients with advanced stage disease. Previously, Chiang et al. demonstrated that histological type was related to the risk of death [3]. Among various types, patients with clear cell carcinoma were reported to have better outcomes than those with serous carcinoma [3]. Nonetheless, there were discrepant findings of relative survival outcomes for patients with clear cell carcinoma and serous carcinoma across different studies [14–16, 25, 26]. For example, Chan et al. [15] and Wei et al. [25] showed that in early-stage EOC, patients with clear cell carcinoma had poorer outcomes than those with serous carcinoma. However, the outcomes of patients with these two histological types in patients with early-stage EOC observed by other investigators were not significantly different [14, 16, 26]. Regarding advanced stages, Schnack et al. [14] and Chen et al. [15] found that patients with clear cell carcinoma had significantly worse outcomes than those with serous carcinoma, which was similar to our findings. Ye et al. [16] observed no significantly different outcomes in patients with these two histological types in advanced-stage EOC. Moreover, taxane-based adjuvant chemotherapy improved the survival of patients with EOC regardless of stage (early or advanced) in the present study. Kajiyama et al. also showed similar results in patients with recurrent EOC [4]. McGuireet al. demonstrated that patients with advanced stage disease administered a taxane plus a platinum agent had significantly better OS and post-recurrence survival than those treated with non-taxane-based chemotherapy [27]. However, whether the taxane-based chemotherapy improves the survival of patients with early-stage EOC is still controversial. Our results demonstrate that taxane-based chemotherapy still provided survival benefits for those with early-stage EOC. The present study provides a useful reference for the importance of treating all patients with ovarian cancer with taxane-based chemotherapy as the frontline therapy.

Stage, a prognostic factor recognized by previous studies [7, 15, 25], was found to have a significant impact on the survival of patients with EOC. Meanwhile, the majority of studies demonstrate that the age at diagnosis is another important prognostic factor [3, 7, 15]. In the present study, we also found that stage and the age at diagnosis were two prognostic factors influencing the survival of patients with EOC. Meanwhile, the residual tumor size was reported to be another important prognostic factor in patients with EOC [28]. Due to a significant lack of information about the status of residual tumor in the database in the present study, conclusions may not be drawn with respect to the impact of residual tumor in the present study.

The strengths of our study were mainly associated with the population-based design, which involved a relatively large sample size, and the representativeness as mentioned before. Although central review for histology was not available, the quality of the histology information in the present study could be relatively high due to the high availability of gynecologic pathologists at most hospitals in Taiwan, especially medical centers. However, detailed information about adjuvant chemotherapies, such as dose administered, or blood test results, such as CA125 levels, was not available in the database. Despite the improvement in the quality of the database, not all the information was complete for each patient. Nonetheless, the present study elucidates the prognostic roles of various factors such as histological type, use of taxane-based chemotherapy, stage, and age at diagnosis. The findings emphasize the importance of histology-oriented research and the treatment selection such as the incorporation of taxane-based chemotherapy, which would be worth further investigation.
